# Undergraduate Research Assistant Leadership for Rigorous, High Quality Research

**DOI:** 10.3389/fpsyg.2019.00474

**Published:** 2019-03-12

**Authors:** Suzanne Wood

**Affiliations:** Department of Psychology, University of Toronto, Toronto, ON, Canada

**Keywords:** undergraduate research experience (URE), undergraduate publication, teaching-research, study drugs, qualitative research and education, teaching faculty

Undergraduate research is an important experiential learning opportunity. Abundant previous work has outlined the benefits of research experiences for undergraduates (e.g., Landrum and Nelsen, 2002; Lopatto, 2004, 2007; although also see Linn et al., 2015). These include refinement of critical thinking skills and clarification of career goals (Seymour et al., 2004; Russell et al., 2007) as well as enhanced motivation to complete academic programs (Graham et al., 2013). Such benefits are found throughout science, engineering, and mathematics (Rodenbusch et al., 2016), particularly in underrepresented groups (Nagda et al., 1988; but see also Lopatto, 2004). The benefits to students seem clear, but what about the benefits to science? Can undergraduate research projects lead to data that is rigorous and worthy of publication? As a teaching stream faculty member at a research-intensive university, my lab consists solely of undergraduate research assistants. Allowing my students to take a leadership role over their projects, particularly projects involving controversial or sensitive topics, has proven to be an effective method, albeit a time intensive one, for generating high quality data.

## Engaging Undergraduates in Publishable Research as Teaching Stream Faculty

Teaching-focused positions have consistently been present at research institutions in relatively small numbers; fairly recently, “teaching stream” positions have become of growing interest in Ontario (Sanders, 2011). These positions are centered around teaching, with service rounding out the responsibilities. While no specific expectations regarding research are detailed for these positions, mixed into teaching is both pedagogical and discipline-specific research, ideally involving undergraduate research assistants. Financial support for research endeavors of teaching stream faculty is provided both at the level of the department (e.g., start-up funds), as well as the division (e.g., Faculty of Arts and Science's Teaching Stream Pedagogical Grants). My department has additionally supported the research endeavors of its teaching stream faculty by providing us with a shared lab space. We are under no pressure to obtain large, federal grants to support our research endeavors (although obtaining funding from the university or outside sources is encouraged).

The intention behind research pursuits of teaching stream faculty is to further education, either by providing undergraduates with authentic research experiences or by systematically examining pedagogical practices. Engaging students in formative educational experiences is the primary outcome for undergraduate research at research universities (Ash Merkel, 2003). While publishing is encouraged for teaching stream faculty, it is generally seen as a mark of educational leadership rather than a necessary step for advancement, as it is viewed in the research stream. Under this framework, teaching stream faculties typically have a great deal of freedom in the type of research pursued with undergraduate research assistants. This freedom allows for potentially greater input from our undergraduates in determining the direction of our research and, ultimately, our publications. Teaching stream faculty can also publish by collaborating with research stream colleagues in the supervision of undergraduates performing work in larger labs, typically in conjunction with graduate students or post-docs (e.g., Abela et al., 2019).

My own lab's research pursuits and publications, however, are entirely fueled by undergraduates. How to best mentor research students, balancing faculty professional directives and student educational goals, is not formally taught at universities, with rare exceptions (e.g., Pfund et al., 2006). In reflecting upon alternatives in setting up a lab, one option would be to structure projects for students before they enter the lab and to guide them through the process. I have chosen to go a slightly different route. While I typically suggest students focus within a certain realm of inquiry (currently, study drug use on campus), I believe the development of their own questions and methodologies serves both as an enriching learning experience, as well as a benefit to their research, more likely leading to publishable data. Indeed, I place a high value on the data generated by my students, as they are heavily invested in the integrity of their results.

## What Can Undergraduates Do?

This is an interesting question, which can be framed in one of two ways. On the one hand, we can consider “what can undergraduates do, anyhow?” From this perspective, we can come up with a list of weaknesses of undergraduate researchers compared to graduate students. There are many. Undergraduates have less experience than graduate students. They have fewer statistical tools under their belts and a weaker understanding of the field of research in which they are engaged. They have less time during the term to devote to research. Undergraduates will also be in a lab for a shorter window of time than a PhD student. This makes having continuity in the lab quite difficult and requires the principle investigator of the lab to engage in the vast majority of hands-on training. It is no wonder that research stream faculty, whose careers are defined primarily by their publications, tend to rely on graduate students and post-docs for producing publishable research. These more senior students and researchers in the lab can also serve as managers of the undergraduates, who may be more highly involved in running experiments rather than designing them (e.g., Weldon and Reyna, 2015), although a subset of research faculty, particularly junior faculty (Ash Merkel, 2003), directly mentor undergraduates, as well (Thiry and Laursen, 2011).

On the other hand, we can frame this question as, “what can undergraduates do that no one else can?” Undergraduate research assistants will have insight into campus culture that could remain otherwise opaque to faculty and graduate students. This insight could help lead a research project related to student behavior down a novel and ultimately fruitful path. Further, having a peer lead a study on a sensitive or controversial topic can put participants at ease, and could arguably lead to more valid data. For example, one of the first projects out of my lab (London-Nadeau et al., 2019), involved my undergraduate research assistants running focus groups on the use of study drugs on campus. Study drugs refer to the use of prescription stimulants by those without a prescription for academic purposes. The undergraduates leading the project entered into my lab with little scientific knowledge about this topic; we spent a good deal of time meeting weekly to discuss papers. We concluded there was very little known about use at our institution and determined that focus groups would be a good starting point.

## Strengths of Undergraduate Research Assistant Leadership

This is where the benefit of research assistant leadership shines through. I could not have led those focus groups, for both ethical and practical reasons. Ethically, there is a decent chance one or more participants would have been students in one of my classes. This would present a conflict in my dual roles as a teacher and a researcher. Knowing about illegal behavior of students in my classes could potentially bias my view of them, which could implicitly affect my evaluation of their performance. This teacher-researcher conflict is of great interest at my university, in regards to not only research on sensitive topics, but also on all pedagogical research projects. While traditional research practices allow us to provide “treatment as usual” in comparison to a new intervention that we believe to be superior, pedagogical research ethics dictate that we cannot withhold a pedagogical intervention we believe to be superior from a subset of our students (MacLean and Poole, 2010 for discussions of the ethics of classroom research; see Healey et al., 2013). While there are still ways to perform controlled, classroom-based pedagogical studies, the standards for ethical approval for these types of studies are higher than for lab-based studies (Martin, 2013 for helpful guides to navigating the ethics review process for classroom research; see Linder et al., 2014). Also, practically speaking, students would have been less likely to be open in a discussion about illegal behavior with a professor present. Even graduate students would have set a more formal tone than having peers running these focus groups. This concern holds true for both teaching and research stream faculty research pursuits involving sensitive topics, in particular.

In consideration of these factors, I needed to rely on my research assistants to be able to direct the conversation and make judgment calls about when to move on to a new topic. They needed to have a firm understanding of issues surrounding study drug use, as well as focus group methodology. The best way to ensure this high level of competency was to have these undergraduates take a leadership role in the development of this project. They understood the previous research surrounding the topic, as well as why each question was being asked, as they had developed the questions, themselves. They were better equipped to think on their feet, which is required for a successful focus group.

After data collection was complete, the undergraduate leading the project learned how to use software specific for coding and analyzing qualitative data. She then trained the other research assistants on the coding process. Writing up the results of this study and submitting for publication was a process that continued after her graduation. However, there was no question she would continue working toward publication. This was her project; she was invested in it. This leadership role directly helped with publication, as this student completed the data analysis and the first draft of the write-up, with guidance. As a pre-tenure professor (technically, “pre-promotion” for teaching stream faculty), my teaching and service commitments would have restricted my ability to complete these last steps independently. I would not expect the same amount of dedication to the project from an undergraduate taking a more ancillary role to the intellectual development of the project.

## Potential Weaknesses of Undergraduate Research Assistant Leadership

There are potential weaknesses of this model worth considering. When given the freedom to design their own projects, undergraduates may gravitate toward a wide range of topics, some of which may be outside of the faculty member's expertise. While supervising students on a range of topics is possible, the most productive mentor relationship would be when the student is working on a topic within the area of expertise of the faculty member. This scenario can be encouraged by the faculty member early on, by guiding the student to explore research possibilities within a specific field through journal club-like readings and discussions. This type of mentorship is similar to the mentor-as-sculptor model discussed and evaluated in another article in this special issue (Holmes and Roberts, in press). This could also be helpful to research stream faculty who are taking on undergraduate students for individual projects. As research stream labs quite practically focus on a subset of questions whose exploration is being funded through grants, the undergraduate researchers in the lab would be best advised to focus on a topic directly related to those broader laboratory pursuits. This can be initially instantiated by the faculty leading the lab, though should also be bolstered by other members of the lab (e.g., graduate students and post-docs) with whom undergraduate researchers tend to spend more time and to develop a mentoring relationship (Behar-Horenstein et al., 2010; Weldon and Reyna, 2015).

## Conclusions

In sum, allowing the time for undergraduates to take ownership over a project can enhance the quality of their research endeavors and facilitate the production of publication-worthy data. To best position undergraduates for success, allocate time early in the term for discussing literature related to the area of expertise of the principal investigator of the lab. Examine recent results, compare current theoretical models, and explore what questions remain unanswered. Remain patient throughout the iterative process of providing feedback on the student's proposed research ideas that will likely need to be reigned in, both in terms of complexity and expense. Once a research question and methodology have been agreed upon, the undergraduate researcher will still require close mentorship throughout the study, but will be in a strong position to take on a leadership role, remain invested in its completion, and produce reliable data, worthy of publication.

## Author Contributions

The author confirms being the sole contributor of this work and has approved it for publication.

### Conflict of Interest Statement

The author declares that the research was conducted in the absence of any commercial or financial relationships that could be construed as a potential conflict of interest.
